# Interactive Ion-Mediated Sap Flow Regulation in Olive and Laurel Stems: Physicochemical Characteristics of Water Transport via the Pit Structure

**DOI:** 10.1371/journal.pone.0098484

**Published:** 2014-05-22

**Authors:** Jeongeun Ryu, Sungsook Ahn, Seung-Gon Kim, TaeJoo Kim, Sang Joon Lee

**Affiliations:** 1 Department of Mechanical Engineering, Pohang University of Science and Technology (POSTECH), Pohang, Gyeongbuk, Republic of Korea; 2 Center for Biofluid and Biomimic Research, Pohang University of Science and Technology (POSTECH), Pohang, Gyeongbuk, Republic of Korea; 3 Neutron Science Division, Korea Atomic Energy Research Institute (KAERI), Daejeon, Republic of Korea; University of California San Diego, United States of America

## Abstract

Sap water is distributed and utilized through xylem conduits, which are vascular networks of inert pipes important for plant survival. Interestingly, plants can actively regulate water transport using ion-mediated responses and adapt to environmental changes. However, ionic effects on active water transport in vascular plants remain unclear. In this report, the interactive ionic effects on sap transport were systematically investigated for the first time by visualizing the uptake process of ionic solutions of different ion compositions (K^+^/Ca^2+^) using synchrotron X-ray and neutron imaging techniques. Ionic solutions with lower K^+^/Ca^2+^ ratios induced an increased sap flow rate in stems of *Olea europaea* L. and *Laurus nobilis* L. The different ascent rates of ionic solutions depending on K^+^/Ca^2+^ ratios at a fixed total concentration increases our understanding of ion-responsiveness in plants from a physicochemical standpoint. Based on these results, effective structural changes in the pit membrane were observed using varying ionic ratios of K^+^/Ca^2+^. The formation of electrostatically induced hydrodynamic layers and the ion-responsiveness of hydrogel structures based on Hofmeister series increase our understanding of the mechanism of ion-mediated sap flow control in plants.

## Introduction

Plants transport sap water through specific porous structures and xylem conduits, which are composed of dead cells. Since the geometrical features of xylem conduits are believed to determine hydraulic conductance, xylem water transport is considered a passive process. Nonetheless, plants can pull water above 100 m in height without any mechanical pumps. They have also survived over hundreds or thousands of years by regulating water transport against unfavorable situations such as severe freezing weather and prolonged drought. Interestingly, water transport in plants can be actively modulated through the xylem (a system of inert pipes).

Flow modulation responds to the small molecules and ion concentration in, and pH levels of, the sap water, and the ion-mediated response in vascular plants is considered a major factor in sap flow regulation. The flow rate of tap water is known to be faster than distilled water in plant stems [1]. Ieperen et al. [2] and Zwieniecki et al. [3] explored hydraulic conductance upon perfusion of plant stems using salt solutions of different ionic concentrations. Subsequent studies [4]–[8] have confirmed the variations in sap flow for a variety of plant species. In particular, enhancement of the ionic effect in embolized stems [9]–[12] is a plausible mechanism for the maintenance of stable water flow under different environmental conditions, compensating for embolism-induced loss of hydraulic conductance.

Due to interest in active flow regulation, previous studies have investigated the effect of ions on sap flow control in terms of a specific cation, K^+^ [2], [3], [5], [8], [13]. However, few studies have been conducted on the effect of other ions on sap flow in plants [12], [14]–[16] from a physiological standpoint. Although the ionic effect in plants has been shown in many experiments, its physico-chemical mechanism remains unclear.

Interestingly, ion-mediated sap flow regulation is a unique process that occurs in dead cells [3], [17]. This suggests that sap flow regulation differs from the biological process that occurs in a living cell, and can be examined from a physicochemical standpoint. In addition, we noted that sap water in plants contain various ions such as K^+^, Ca^2+^, Mg^2+^, and Na^+^ for optimizing water transport under different environmental conditions [18]. Thus, we considered the interactive effects of ion mixtures on water flow regulation. To characterize ion-responsiveness in vascular plants, we investigated the interactive effects of the type and composition of ions on pit membranes and sap flow by systematically varying the molar ratios of K^+^ to Ca^2+^. These two ions are the major cations in xylem sap water [12], [18]–[20] and are closely associated with water transport in plants. For example, K^+^ facilitates water flow by controlling evaporation rates from stomata via guard cell regulation [21] and Ca^2+^ by modulating cell wall structure, stomatal aperture and aquaporin stimulation [22], [23].

Synchrotron X-ray and neutron imaging methods allow us to directly monitor ion-responsiveness in the stems of vascular plants in a noninvasive and nondestructive manner. Unlike perfusing solutions, these imaging techniques can be employed to characterize sap flow without exerting an artificial positive pressure, which could induce morphological changes in the plant nanostructure [8], [24]. Synchrotron X-ray imaging is advantageous for investigating both the xylem structure of vascular plants and the sap flow in individual xylem levels with sufficient spatial and temporal resolutions [25]–[27]. On the other hand, the neutron imaging method is usually operated with relatively low flux for an expanded field of view (FOV) to investigate ionic solution uptake over a longer time scale [28]. In addition, the neutron imaging method can detect small changes in light elements such as hydrogen, allowing for *in situ* visualization of water transport in plants [28]–[33].

In this report, to characterize ion-mediated sap flow regulation in vascular plants from a physicochemical standpoint, the interactive ionic effects of K^+^ and Ca^2+^ on sap flow in olive and laurel stems were examined using synchrotron X-ray and neutron imaging methods. Our experimental results on the interactive ionic effects increase our understanding of structural changes in pit membranes and the physicochemical mechanism of active sap flow regulation in xylem conduits.

## Materials and Methods

### Plant materials and experimental conditions

We selected olive (*Olea europaea* L.) and laurel (*Laurel nobilis* L.) as test samples because they are known to be highly sensitive to ions in sap water [5], [8]. Olive and laurel stems were excised at lengths of 15 and 25 cm for X-ray and neutron imaging experiments, respectively, to characterize sap water distribution and regulation in their stems (excluding other factors affected by roots and leaves). Given that the ionic effect increases up to a length of 12 cm and then remains constant for longer stems [11], the length of the excised stems was greater than the critical length to maximize the ionic effects on hydraulic conductance. The bottom ends of the excised stems were dipped in reservoirs containing ionic solutions of different K^+^/Ca^2+^ ratios, and the uptake process was monitored using synchrotron X-ray and neutron imaging techniques. During the ascent of ionic solutions, the temperature and humidity of environmental air were maintained at 19±1°C and 95±1% relative humidity (RH). During the experiments, the photosynthetic active radiation was 200 µmol m^−2^ s^−1^.

Although the physiological ionic ratio of K^+^/Ca^2+^ ranges from 3 to 10, depending on environmental conditions [12], [18], [20], we used ionic solutions with K^+^/Ca^2+^ ratios of 0.01, 1, and 100 to characterize the interactive ion-response in sap flow. The total concentration of ions was fixed at 50 mM to explore the effects of ion type and ion composition on hydraulic conductance and to maximize the subsequent ionic effect. Ion-mediated variations of hydraulic conductance are known to be concentration-dependent, but the ionic effect is generally saturated at 20 mM to 50 mM KCl [3], [8]. Although a total concentration of 50 mM is greater than the normal xylem sap osmolality in laurel stems, which ranges from 20 to 50 mOsm kg^−1^ and corresponds to 10 to 25 mM KCl [34], this concentration may be biologically harmless. Ionic solutions with varying K^+^/Ca^2+^ ratios were produced by mixing 0.5 mM KCl and 49.5 mM CaCl_2_, 25 mM KCl and 25 mM CaCl_2_, and 49.5 mM KCl and 0.5 mM CaCl_2_, respectively.

### Synchrotron X-ray micro-imaging and neutron imaging techniques

Synchrotron X-ray micro-imaging experiments were performed at the beamline 14C from the Photon Factory (PF), KEK (High Energy Accelerator Organization), Tsukuba, Japan. Using a white (full-energy spectrum) X-ray beam with an energy of 70 keV, X-ray images with a field of view (FOV) of 1.5×1.5 mm were obtained with a spatial resolution of ∼10 µm. X-ray images were recorded consecutively at 3-min intervals. To minimize radiation damages, a mechanical shutter and attenuating plates were employed to expose the sample plants to attenuated X-ray beam only when we captured X-ray images. Iopamidol solution was mixed with the ionic solutions as an X-ray contrast agent to clearly visualize the uptake process of ionic solutions in plant stems.

The neutron imaging experiments were conducted at the neutron radiography facility (NRF) of HANARO (30 MW), Korea Atomic Energy Research Institute (KAERI), Korea. A thermal neutron beam with a mean energy of 14 meV was collimated and detected using a ^6^LiF/ZnS:Ag scintillator (PSI, Switzerland) and a CCD camera attached with a zoom lens (f 2.0, 135 mm). The thermal neutron flux was 5.07×10^6^ neutrons cm^−2^ s^−1^ and the effective beam size was approximately 350×450 mm. The FOV of neutron radiography was 11.3×11.7 cm and each image was expressed at 1340×1300 pixels with a spatial resolution of 50 µm. Neutron images were recorded consecutively for several hours and the total image acquisition time for one image was 41.5 s, which consisted of 40-s exposure and 1.5-s readout. Deuterium oxide (D_2_O), which has physiological properties similar to those of H_2_O in plants, was employed as a contrast agent in neutron imaging experiments [35]–[37]. Despite long-term expose to D_2_O, we observed no wilting and no morphological changes in the test plants. This implies that D_2_O application as a contrast agent does not perceptible damages to the test plants.

### Image processing for X-ray and neutron images

The effect of temporal fluctuations in the incident beam intensity on the transmission images was corrected using Octopus software (http://www.xraylab.com). Flat-field correction was adopted to eliminate the CCD dark current noises and the heterogeneity of the beam profile. The corrected image (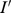) was calculated as follows:
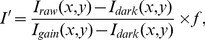
(1)where 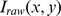 is the raw image, 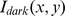 is the image taken with neither beam nor test sample, 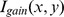 is the image captured without the sample under the same experimental condition, and 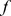 is a scaling factor. The calculations were performed pixel-wise at the lateral and vertical positions *x* and *y*, respectively. A median filter was adopted to remove noises embedded in the corrected images.

Although the excised stem segments were fixed, the exact positions of the sample stems could change slightly during long-term observations in neutron imaging experiments. This slight change in sample position was corrected by 2D translation within a fraction of a pixel [38]. After this image-alignment process, the corrected initial image (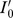) was divided by the subsequent images (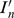) at time 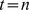, as follows:
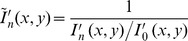
(2)This procedure was performed to enhance the sensitivity of detection of light intensity variations during the experiment and to quantitatively compare the uptake behaviors of ionic solutions. The enhanced X-ray and neutron images (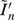) were color-coded into 32 levels and processed with the jet colormap provided by Matlab, respectively.

An 80×30-pixel region of interest (ROI) was selected to quantitatively analyze the X-ray images. The intensity values in the ROI were then averaged and the results in the consecutive images were compared. To show the relative variations in ionic water uptake effectively, normalized intensity (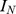) was determined from the pixel intensities of each image at a time 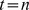 to the corresponding pixel intensities of the first image at 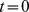 (see Eq. (3)).



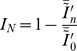
(3)where 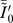 is the averaged intensity value in the ROI of the initial image and 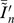 represents the averaged intensity value in the ROI of time-consecutive images. 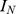 quantitatively shows the change in X-ray absorption. In other words, an increase in the average variations of 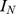 directly corresponded to an increase in ionic solution uptake to replace water already present in a plant.

The relationship between the normalized neutron images and the thickness variation is based on the Beer-Lambert attenuation law [37]. The excised stem segment was assumed to be partitioned into H_2_O, D_2_O, and dry biomass fractions. The local attenuation coefficient 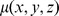 was assumed to be constant [39]. At time 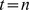, the intensity 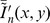 of a neutron beam passing the test sample with a thickness 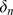 in the 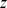-direction was approximated as:
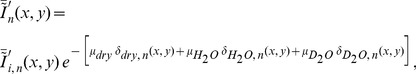
(4)where 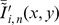 is the incident beam intensity distribution and 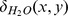,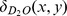, and 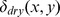 indicates the hypothetical thickness of H_2_O, D_2_O, and dry biomass fractions, respectively. Using the initial image (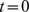) as a reference, the 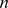 ^th^ image was normalized as 
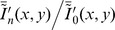
. In addition, 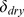 and 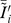 were assumed to be constant during the experiment. The decrease in H_2_O corresponded to an increase in D_2_O, which can be expressed as 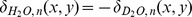. The temporal variation in D_2_O thickness (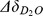) in the 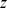-direction up to time 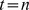 at any position 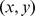 in the camera plane can be quantified using the following measured beam intensities:
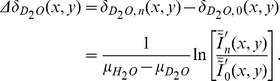
(5)The thickness 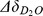 corresponded to ionic D_2_O uptake. The linear attenuation coefficient usually depends on the neutron-beam spectrum. Given that the neutron beam consists of a set of neutrons with different energies, the linear attenuation coefficient is usually increased by integrating over different neutron energies. The measured linear attenuation coefficient of H_2_O at NRF[40] was 0.3029±0.0002 mm^−1^. The linear attenuation coefficient of D_2_O was estimated to be 0.0303 mm^−1^.

A threshold value corresponding to a specific neutron absorption intensity was applied to the neutron images captured in this study to evaluate the increased level (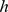) of ionic solutions. First, intensity values were averaged along the horizontal 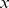 direction of the ROI for each stem. The threshold value was then applied consistently to determine the height of the diffusional interface (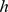) between H_2_O and the ionic D_2_O among the horizontally averaged intensity values (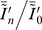). When the value of 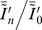 was below the threshold value, the pixel information was thought to represent the contents of D_2_O. Changes in 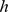 with time were quantitatively compared at different K^+^/Ca^2+^ ratios.

## Results and Discussion

**Figure 1:**
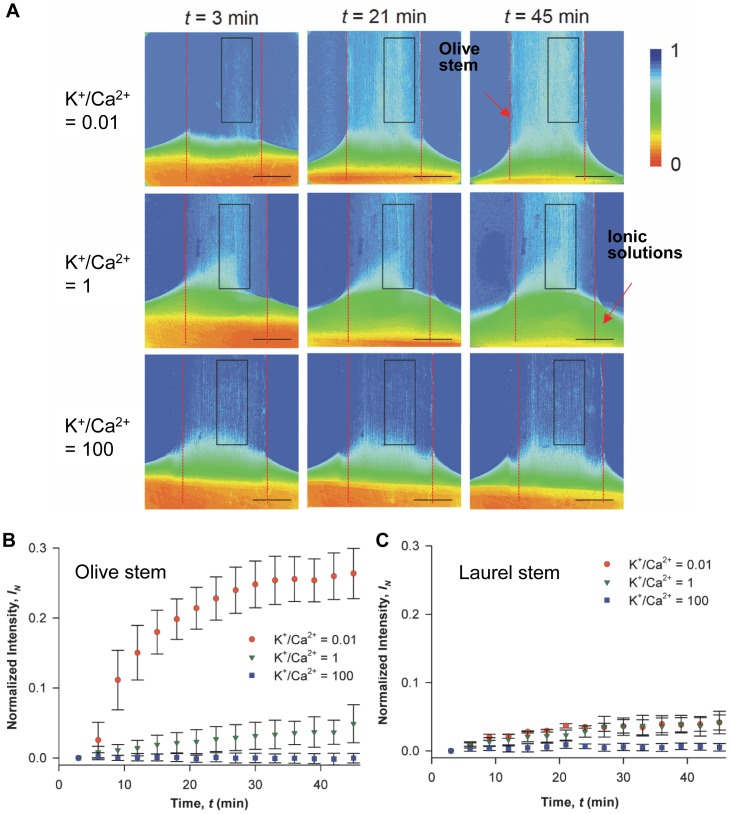
Synchrotron X-ray micro-imaging of the ascent of water containing K^+^ and Ca^2+^ in xylem vessels. **A**) Typical X-ray images showing temporal changes in the spatial distribution of ionic solution uptake mediated by varying K^+^/Ca^2+^ ratios from 0.01, 1, to 100 in the excised olive stems. Synchrotron X-ray imaging was employed to observe ionic water uptake directly through the xylem conduits of olive stems with high temporal and spatial resolution. Excised stems with a height of 15 cm took up ionic solutions with different K^+^/Ca^2+^ ratios under the same total ion concentration. Intensity-based colored figures in each column show qualitative information regarding water ascent at different ionic ratios: K^+^/Ca^2+^  =  0.01 (1^st^), 1 (2^nd^), and 100 (3^rd^), respectively. Light blue color represents the ascent of multi-ionic solutions mixed with iopamidol as a contrast agent. The X-ray image of each column was captured at 3, 21, and 45 min, respectively. Scale bar represents 500 µm. Temporal variations in the normalized X-ray absorption intensities in the region of interest for **B**) olive stems and **C**) laurel stems. Orange circles denote K^+^/Ca^2+^  = 0.01; green rotated triangles denote K^+^/Ca^2+^  = 1; purple rectangles denote K^+^/Ca^2+^  = 100. The normalized intensity values directly represent ionic solution uptake. These figures were derived from three repeated experiments (n = 3) and error bars indicate standard deviations.

The spatial distribution of the ionic solutions of the same concentration (50 mM) but different K^+^/Ca^2+^ ratios in excised olive stems was visualized using synchrotron X-ray imaging (Fig. 1A). The images were selected at time 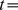
3, 21, and 45 min. The first, second, and third rows corresponded to K^+^/Ca^2+^ ratios of 0.01, 1, and 100, respectively. In particular, the replacement of sap water in the xylem conduits with newly introduced ionic solution could be noninvasively investigated using iopamidol as a nonionic X-ray contrast enhancer. The degree of X-ray absorption change was expressed by color in Fig. 1A with a scale bar on the right ranging from a high increase in X-ray absorption (red) to no change (dark blue). Therefore, the increase in X-ray absorption (and thus the color shift from dark blue) illustrated an increase in ionic solution with iopamidol. Synchrotron X-ray images show local inhomogeneity in the X-ray absorption, as shown in square boxes of Fig. 1A, which represent the ROI in xylem conduits rapidly filling with newly introduced ionic solutions. In the ROI, different xylem conduits pulled-up the ionic solutions at varying speeds.

In Figs. 1B and 1C, the overall variation in ionic solution uptake at different K^+^/Ca^2+^ ratios was compared using intensity-based analysis of X-ray images. Normalized intensity (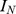) was determined from the pixel intensities of each image at a time t = t_1_ to the corresponding pixel intensities of the first image at 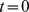 (Eq. (3)), which quantitatively shows the change in X-ray absorption. An increase in the average variations of 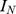 directly corresponded to an increase in ionic solution uptake to replace existing water in a plant. The 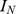 value increased at lower K^+^/Ca^2+^ ratios for both systems. The overall change in the normalized intensity (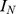) at K^+^/Ca^2+^  = 0.01 or 1 was more rapid than at K^+^/Ca^2+^  = 100 in olive stems in a relatively short time (45 min).

**Figure 2:**
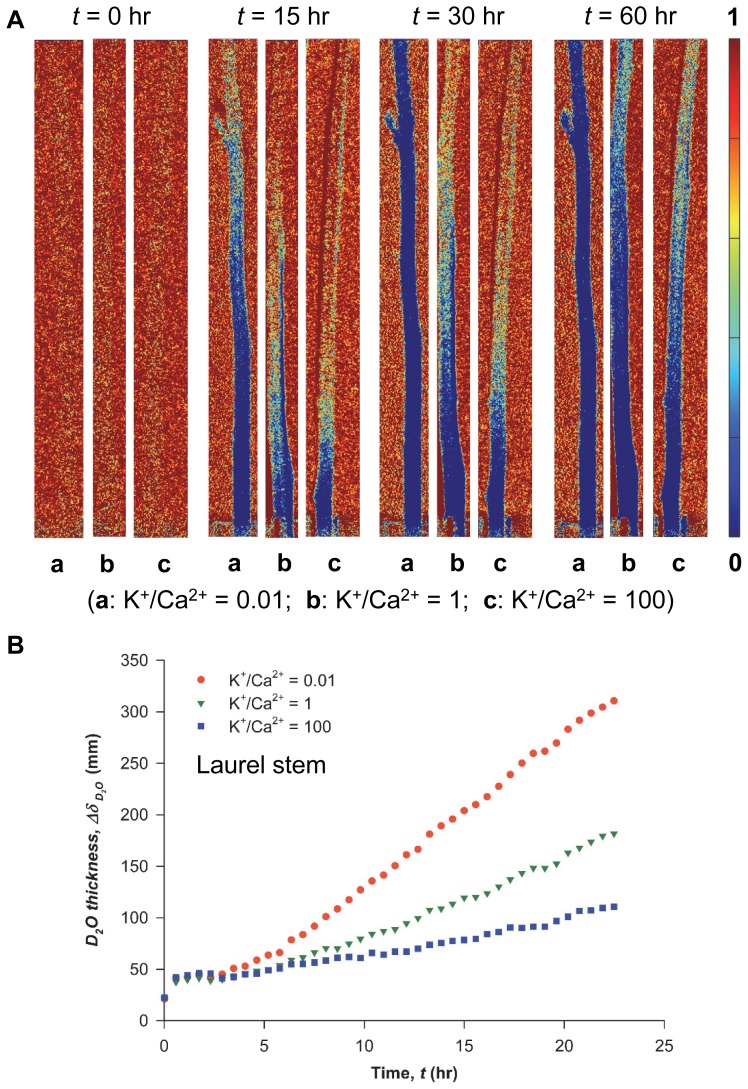
Neutron imaging of ionic solution uptake with different K^+^/Ca^2+^ ratios in plant stems. **A**) Neutron images of ionic solution uptake with K^+^/Ca^2+^ ratios of 0.01, 1, and 100 in the excised laurel stems at 0, 15, 30, and 60 h after dipping in the ionic D_2_O solution. Ionic D_2_O uptake in the excised laurel stems was directly observed using neutron radiography. Each figure provides qualitative information on water ascent at different ionic ratios (K^+^/Ca^2+^  = 0.01 (left), K^+^/Ca^2+^  = 1 (middle), and K^+^/Ca^2+^  = 100 (right)) at 0, 15, 30, and 60 h, respectively. The average diameter of stem segments was approximately 5 mm. Scale bar represents 5 mm. **B**) Temporal variations in ionic D_2_O thickness in the laurel stems for 23 h. Orange circles denote K^+^/Ca^2+^  = 0.01; green rotated triangles denote K^+^/Ca^2+^  = 1; purple rectangles denote K^+^/Ca^2+^  = 100.

To further investigate the uptake of ionic solutions over longer time scales, neutron imaging techniques were employed with laurel stems. Laurel responded to ions more slowly than olive. Figure 2A shows the time-averaged ionic water uptake by tracing the spatial distribution of D_2_O containing varying K^+^/Ca^2+^ ratios in excised laurel stems at 0, 15, 30, and 60 h after dipping in ionic D_2_O. The degree of neutron transmission was also color scaled as shown in the scale bar on the right of Fig. 2A. In Fig. 2A, blue shift represents better neutron transmission compared with the first image, while red shift represents no transmission change. Therefore, blue color corresponds to ionic D_2_O solution uptake and replacing H_2_O present in the stems. Assuming the stem samples showed the same initial water potential, the neutron images clearly demonstrated that when the K^+^/Ca^2+^ ratio was 0.01, the ionic D_2_O uptake was much faster than at higher K^+^/Ca^2+^ ratios of 1 or 100 for a longer time scale in the excised laurel stems. The modulation of water uptake according to K^+^/Ca^2+^ observed by neutron imaging for an extended time period (65 h) was similar to the results obtained by synchrotron X-ray imaging for short time durations (50 min). These results suggest that the interactive effect of different K^+^/Ca^2+^ ratios at a fixed concentration (50 mM) has a considerable influence on the water uptake process, irrespective of plant species or time scale.

Temporal characteristic variations in ionic D_2_O thickness (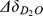) affected by the K^+^/Ca^2+^ ratio in the laurel stem at the field of view quantitatively show the interactive role of monovalent and divalent cations during the uptake of ionic solutions (Fig. 2B). The ionic D_2_O thickness (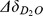) was estimated from the ratio of the neutron absorption intensity at time 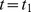 of each image to that of the first image at 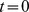 (Eq. (5)), which corresponded to ionic D_2_O uptake. The contribution of K^+^/Ca^2+^ ratios on the uptake of ionic solution in laurel stems was insignificant during the initial 5 hr. However, the 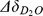 increased significantly over time at lower K^+^/Ca^2+^ ratios. The ionic solution uptake rate was in the order of the K^+^/Ca^2+^ ratio of 0.01>1>100. In particular, when the K^+^/Ca^2+^ ratio decreased by 100-fold, the uptake rate of the ionic solutions in the laurel stems increased by approximately twofold. Changes in sap water uptake efficiency according to K^+^/Ca^2+^ based on neutron imaging for an extended time (up to 60-h time scale) was similar to the results obtained from synchrotron X-ray imaging for a short time (50-min time scale). The interactive effect of different ionic compositions (K^+^/Ca^2+^) at a fixed concentration (50 mM) strongly affected the water uptake process irrespective of plant species and time scale.

**Figure 3:**
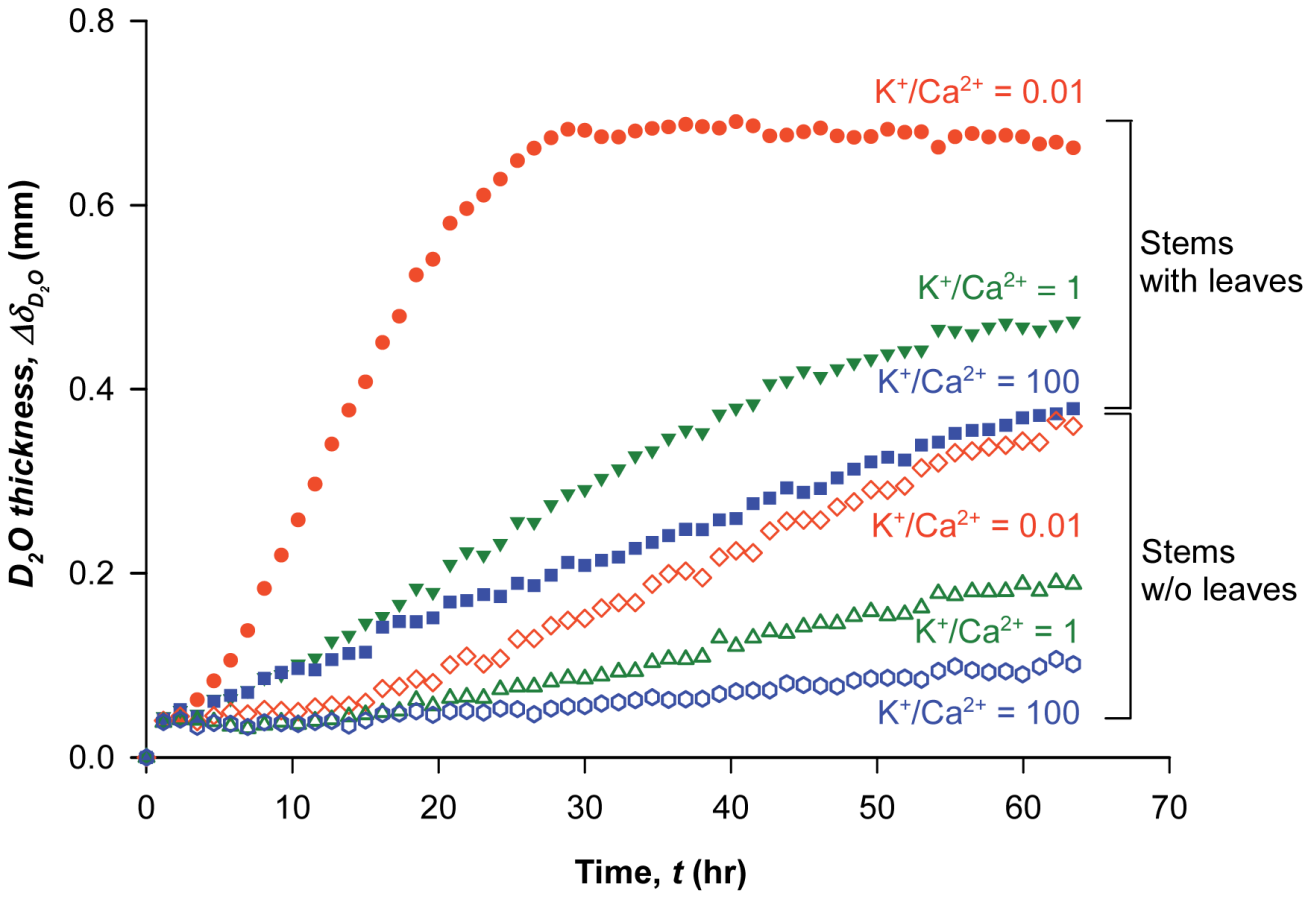
Physicochemical process of ion-mediated sap flow regulation. Effect of K^+^/Ca^2+^ ratios on the variations of ionic D_2_O thickness in the laurel stems with and without leaves for 64 h. Temporal variations in ionic D_2_O contents in the region of interest for the excised laurel stems with leaves (closed symbol) and without leaves (open symbols) were measured using neutron imaging technique for 64 h. Closed orange circles and open orange diamonds denote K^+^/Ca^2+^  = 0.01; closed green rotated triangles and open green triangles denote K^+^/Ca^2+^  = 1; and closed purple rectangles and open purple hexagons denote K^+^/Ca^2+^  = 100.

To determine whether ion-mediated water flow modulation was independent of that driven by transpiration in leaves, the effects of different K^+^/Ca^2+^ ratios were investigated in laurel stems with and without leaves. Fig. 3 shows temporal variations in ionic D_2_O thickness (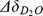) along the excised stems of laurel samples with and without leaves for 65 h. The thickness 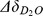 corresponded to ionic D_2_O uptake (see Materials and Methods). On average, the uptake rates of the ionic solutions through leafless stems were twofold lower compared with stems with leaves that transpire under relatively high humidity conditions (95±1%). In addition, the ascent of ionic D_2_O was more significant and faster at the lowest ionic ratio (K^+^/Ca^2+^  = 0.01). The uptake rate of ionic solution was in the order of the K^+^/Ca^2+^ ratio of 0.01>1>100 for both systems with and without plant leaves. The varying cationic ratios synergistically regulated sap water transport in plants, irrespective of the existence of leaves.

**Figure 4:**
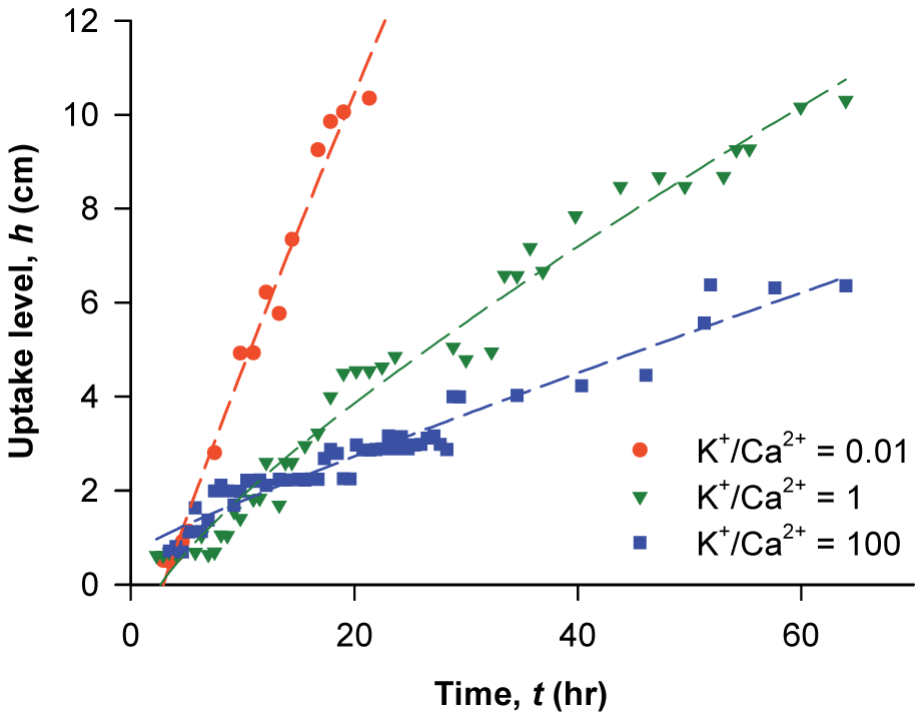
Effect of K^+^/Ca^2+^ ratios on the uptake level of ionic solution. Temporal variations in the increase in ionic solutions with three K^+^/Ca^2+^ ratios in the excised laurel stems and curve-fitted with 2-D diffusion-convection model. Variations in the increased K^+^/Ca^2+^ ratios were quantified using a neutron imaging technique for 64 h. Orange circles denote K^+^/Ca^2+^  = 0.01; green rotated triangles denote K^+^/Ca^2+^  = 1; purple rectangles denote K^+^/Ca^2+^  = 100.

The increase in ionic solution (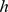) was measured by consistently applying the same threshold value corresponding to a specific neutron absorption intensity for the neutron images (see Material and Methods). Changes in 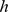 with time for other ionic solutions of different K^+^/Ca^2+^ ratios were then compared quantitatively (Fig. 4). The ionic effect is localized at pit membranes [3], [17], which limit hydraulic conductance in xylem [41]–[43]. Thus, we assumed that the axial transport of ionic solution corresponded to radial transport through pit membranes in xylem.

The uptake of ionic solutions in our experiments was due to the convective flow under hydrostatic pressure differences driven by leaf transpiration and the diffusion driven by concentration differences between the ionic solutions and sap water already present in plants. Therefore, this can be modeled using two-dimensional diffusion-convection, which can be expressed as the following Eq. (6) [44].
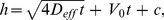
(6)where 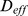,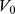, and 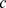 are the effective diffusion coefficient, the convective velocity, and a constant, respectively. The water level 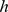 of an ionic solution was curve-fitted by Eq. (6), and the fitted curves (dotted lines in Fig. 4) can be used to determine 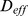,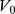, and 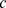 in each case. The diffusion coefficients 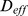 were determined to be 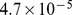
cm^2^/sec, 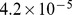
cm^2^/sec, and 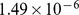
cm^2^/sec when the K^+^/Ca^2+^ ratios were 0.01, 1, and 100, respectively. The value of 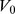 was in the order of 10^−5^ cm/s and the Péclet number was 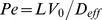
<<1, where 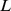 is the characteristic length. This suggested that diffusion was dominant over convection for all K^+^/Ca^2+^ ratios tested in this study. We can assume that the difference in initial water potential in all stem samples was negligible. The values of 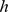 were well-matched using curve-fitting with adjusted R^2^ values of 0.98, 0.97, and 0.94, respectively.

The diffusion through pit membranes differed from that through homogeneous solution because it was influenced by the structural features of pores, such as size, shape, and connectivity. The 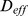 for diffusion through a porous media was estimated based on the diffusion coefficient of ions in a dilute solution (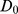), the porosity (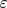), and the tortuosity (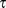), as follows [44]:
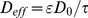
(7)The change in porous structure used for water transport can be estimated from 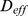 obtained using the aforementioned curve-fitting. The change in 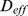 at different K^+^/Ca^2+^ ratios corresponded to the ratio of porosity to tortuosity (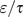) of the pit membrane. From the fitted curves, the 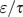 ratio of the pit membrane was observed to increase by approximately 12% at the ionic ratio of K^+^/Ca^2+^  = 0.01, compared with that at K^+^/Ca^2+^  = 1. Conversely, the ratio 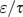 decreased by 96% at K^+^/Ca^2+^  = 100, compared with that at K^+^/Ca^2+^  = 1. These results suggested that the effective porosity of pit membranes decreased sap flow as the K^+^/Ca^2+^ ratio increased. Since the water flow through pit membrane plays a major role in determining the overall water transport through xylem conduits [43], we hypothesized that dynamic changes in the morphological structure of pit membrane induced by ions would be important for active water flow regulation in vascular plants.

To characterize the mechanism of interactive ionic effects on effective structural changes of pit membranes, we should consider the chemical compositions of pit membranes and the ion-structure interaction. Pit membranes are composed of coextensive cellulose-hemicellulose networks forming a fiber composite material as a primary cell wall structure [45]. Pectic polysaccharide is a water-retentive, shear-resistant matrix in a primary cell wall structure, but whether this hydrogel-like constituent is present in intervessel pit membranes and its function on the ionic effect remain unclear.

**Figure 5:**
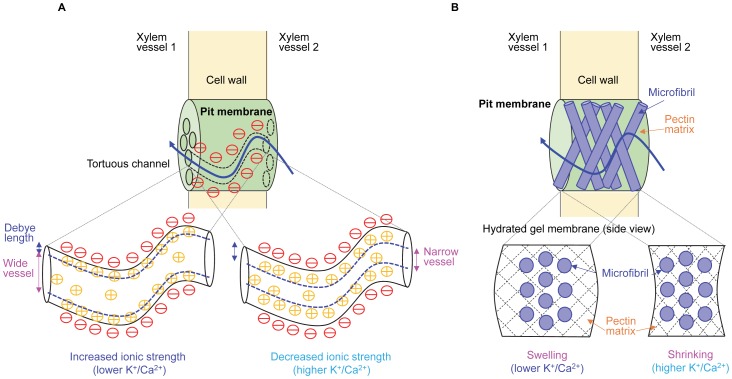
Putative physicochemical models for ion-mediated sap flow regulation in vascular plants. Schematic illustrations of **A**) the electrostatically induced hydrodynamic layer formation model and **B**) the ion-responsive hydrogel model to explain ion-mediated sap flow regulation in vascular plants. In the electrostatically induced hydrodynamic layer formation model, the ionic ratios affect the effective porous structure for control of water transport via the pit membrane. A relatively thin Debye layer formed at lower K^+^/Ca^2+^ ratios, making a relatively wider flow path in the pit membrane, while a relatively thick Debye layer induced a narrow flow path at higher ionic ratios. In the hydrogel model and at lower K^+^/Ca^2+^ ratios, pectins are swollen in the pit membrane with increased porosity based on Hofmeister series. This ion-mediated structural change seems to contribute to the increased flow rate through xylem vessels. At higher ionic ratios, pectins shrunk in the pit membrane with relatively decreased porosity. The effective structural changes may reduce the flow rate.



Taking into account the controversy over the existence of pectins in pit membranes of plants [46]–[49], two scenarios can explain ion-mediated variations in the effective porosity of pit membranes (Fig. 5). First, the effective porosity of pit membranes can be affected by electrostatically induced hydrodynamic layers on the pit wall [50], [51]. The negatively charged surface of hydrated pit membranes [8] attracts cations from ionic solutions, creating the Debye layer near the surface. Since the concentrated cations in the Debye layer give rise to drag, the Debye length (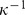) affects the effective pore size through which sap water passes. The flux density (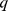) can be expressed as follows:

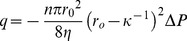
(8)where 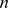 is the number of pores per unit surface area, 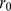 is the intrinsic radius of pore, 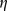 is fluid viscosity, and 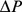 is the pressure difference per unit pore length. The Debye length 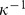 is reciprocally proportional to the root of ionic strength (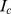), and can be obtained as follows:



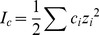
(9)where 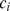 is the molar concentration of ion 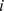 and 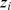 is the ion valence. Therefore, even at the same concentration, the 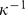 decreases and 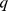 of sap water increases as the K^+^/Ca^2+^ ratio decreases (Fig. 5A). This mechanism based on electrostatic interactions suggests that ion types and ionic compositions are important factors in ion-mediated flow regulation in vascular plants.

Second, by assuming that pit membranes are composed of a pectin-based hydrogel, the swelling and shrinking phenomena of hydrogels [1], [3], [13] can be used to characterize the interactive ion-mediated variation in the pore size of pit membranes, and thus flow regulation in vascular plants. In particular, the ion-specific Hofmeister series [52] can be used to explain the effects of different ion types and ionic ratios. According to the series, salting-out K^+^ causes shrinking of the hydrogel, while salting-in Ca^2+^ induces swelling. Thus, a lower K^+^/Ca^2+^ ratio is expected to induce swelling in the hydrogel structure, whereas a higher K^+^/Ca^2+^ ratio causes relatively less swelling or shrinking (Fig. 5B). Shrinking of the hydrogel structure could decrease the porosity of pit membranes. Conversely, the swelling of hydrogel increases the porosity of pit membranes. Therefore, the flow rate of ionic solutions increased at lower K^+^/Ca^2+^ ratios. The effects of structural variation in a hydrated gel matrix on the ion-responsive flow regulation in vascular plants can be used to characterize ionic effects.

In conclusion, we examined the ion-responsiveness of water transport in olive and laurel stems to increase our understanding of the mechanism of active water flow regulation in two representative vascular plants. Different ion types significantly affected water transport in xylem conduits. Sap flow rate decreased with an increasing K^+^/Ca^2+^ ratio, while a decrease in the K^+^/Ca^2+^ ratio accelerated the ascent of ionic solution. This interactive ionic effect is a common type of systematic flow regulation in vascular plants, irrespective of the plant species. The active ion-mediated flow regulation in the stem of a vascular plant may result from structural changes in pit membranes. The effective structural changes mediated by ions can be explained by the formation of electrostatically induced hydrodynamic layers and the ion-responsiveness of hydrogel structure based on Hofmeister series. The interactive ionic effects from a physicochemical standpoint describe the role of ion-responsiveness during water flow regulation in vascular plants.
